# Neuronal Ceroid Lipofuscinosis: The Multifaceted Approach to the Clinical Issues, an Overview

**DOI:** 10.3389/fneur.2022.811686

**Published:** 2022-03-11

**Authors:** Alessandro Simonati, Ruth E. Williams

**Affiliations:** ^1^Departments of Surgery, Dentistry, Paediatrics, and Gynaecology, School of Medicine, University of Verona, Verona, Italy; ^2^Department of Clinical Neuroscience, AOUI-VR, Verona, Italy; ^3^Department of Children's Neuroscience, Evelina London Children's Hospital, London, United Kingdom

**Keywords:** neuronal ceroid lipofuscinosis, NCL clinical features, NCL pathogenetic mechanisms, NCL treatments, NCL review

## Abstract

The main aim of this review is to summarize the current state-of-art in the field of childhood Neuronal Ceroid Lipofuscinosis (NCL), a group of rare neurodegenerative disorders. These are genetic diseases associated with the formation of toxic endo-lysosomal storage. Following a brief historical review of the evolution of NCL definition, a clinically-oriented approach is used describing how the early symptoms and signs affecting motor, visual, cognitive domains, and including seizures, may lead clinicians to a rapid molecular diagnosis, avoiding the long diagnostic odyssey commonly observed. We go on to focus on recent advances in NCL research and summarize contributions to knowledge of the pathogenic mechanisms underlying NCL. We describe the large variety of experimental models which have aided this research, as well as the most recent technological developments which have shed light on the main mechanisms involved in the cellular pathology, such as apoptosis and autophagy. The search for innovative therapies is described. Translation of experimental data into therapeutic approaches is being established for several of the NCLs, and one drug is now commercially available. Lastly, we show the importance of palliative care and symptomatic treatments which are still the main therapeutic interventions.

## Introduction: Historical Notes

The Neuronal Ceroid Lipofuscinoses (NCL) are neurodegenerative disorders, mostly of childhood onset. They form a heterogeneous group of lysosomal storage diseases (LSD) mainly affecting brain and retina (1). They are genetic disorders, and the first description of putative juvenile NCL was of four siblings in Norway with progressive visual loss, cognitive decline, seizures and premature death. This report remained unnoticed until 150 years later (2).

Cases of progressive visual loss with cognitive decline of infantile and/or childhood onset and a fatal outcome were grouped around the turn of the 19^th^ century under the term “amaurotic familial idiocy”, coined by the American neurologist Sachs (3), and included the ocular manifestations described by the British ophthalmologist Tay (4). Several familial cases were described from different European countries. The contribution of neuropathology further characterized this group of diseases by describing the topography of brain abnormalities and the selective involvement of the cerebral and cerebellar cortices and of subcortical gray nuclei (5–9). Adult onset cases were reported by Kufs (10). The common appearance of swollen cerebral neurons (as well as retinal ganglion cells), whose topology was distorted because of the cytoplasmic engulfment with granular materials with similar staining properties was also described (11). Batten (12) and Spielmeyer (8) went on to outline some differences in the features and distribution of storage material between these entities, allowing division of the amaurotic familial idiocies into different clinical entities with different genetic backgrounds (13), specific biochemical properties (14, 15), selected neuropathological features (16), and eventually genetic markers toward the end of the last century. This was the beginnings of the classification and nosography of so-called storage diseases.

NCL are currently grouped under two major eponyms, Batten disease and Kufs disease. Batten disease refers to childhood NCLs, regardless the age of onset, whereas the term Kufs disease is assigned to the two major phenotypes of adult onset NCL [Kufs A and B (17)]. NCL definition relies on pathological criteria: the presence of autofluorescent lipofuscin and the characteristic cytosomes. Zeman and colleagues introduced the term “Neuronal Ceroid Lipofuscinosis” based on the histochemical and ultrastructural features (18). The identification of such markers allowed the NCLs to be further characterized and distinguished from other “amaurotic familial idiocies”, such as gangliosidoses (19).

Intracytoplasmic accumulation of autofluorescent material is a pathogenetic hallmark of the NCLs, and it is ascribed to the abnormal storage of ceroid, a pathologically derived material, with similar biochemical properties to lipofuscin, the “aging pigment” (20). The abnormal storage material is embedded within lysosomes, and its ultrastructural features probably reflect the chemical composition of the storage and the related aggregates (16). The biochemical composition of the storage is still only partially defined. A main component (subunit c of the mitochondrial ATP synthase) accumulates in the late infantile variants and in juvenile onset NCL; sphingolipid activating proteins (Saposins A and D) are enriched in two infantile onset forms (21, 22). Interestingly these components are detected in CNS tissue only, and they are not present in peripheral tissues, where the storage can be detected in several cell types ultrastructurally.

The recognition of lysosomal involvement in the NCLs was followed by a huge research effort aiming to disentangle the pathogenetic mechanisms and to shed light on the cellular pathways and processes which are affected (see Section The Research Contribution to Knowledge).

The identification of lipopigment storage ultrastructurally became the main diagnostic tool for NCL, and cerebral biopsies represented the favored diagnostic approach. Evidence that storage material could be detected in readily accessible extra-neural tissues (such as skin, blood lymphocytes, skeletal muscle) led to an important shift from CNS to peripheral biopsies, a safer and more rapid pathway to diagnosis (23). Peripheral neurons, such as intramural ganglionic neurons of rectal mucosa, were also utilized for diagnostic purposes (24, 25). The impaired function of these cells was associated with gastrointestinal problems, including abdominal pain, constipation, and altered bowel motility in NCL patients. The involvement of the enteric nervous system was recently investigated in three NCL mouse models, which showed both impaired enteric functions and histological and ultrastructural findings consistent with neuronal loss and storage accumulation (26).

**Figure 1:**
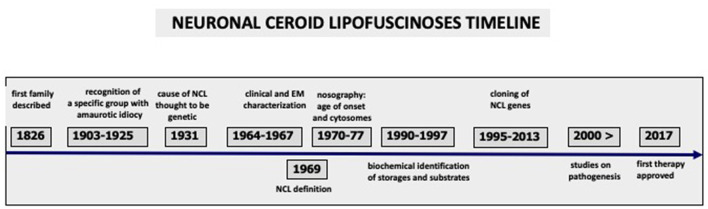
Major steps and achievements during nearly two centuries of Neuronal Ceroid Lipofuscinoses history are outlined, starting from the earliest clinical description to the present research which led to the first FDA/EMA approved treatment.

Based on clinical and pathological criteria, the NCLs were classified according to age of clinical onset and the ultrastructural features of the cytosomes. Their nomenclature was modified over time to include newly recognized variants [for example, infantile and early juvenile] and the adult form (16, 27, 28). Such classification has been valid for about four decades, and it is still useful in a clinical setting (Table 1). It has helped to target molecular genetic diagnostic investigations. The identification of cytosomes in the peripheral tissues of unusual cases, where the clinical phenotypes is not entirely consistent with known NCL genetic types, may lead to molecular analysis using NCL panels, sometimes obtaining diagnostic confirmation at molecular level (29). Major steps in NCL history are summarized in Figure 1.

**Table 1:** NCL of childhood onset: clinical classification and major diagnostic procedure.

**Clinical form**	**Age of onset**	**Disease**	**Gene**	**Diagnosis**	**Major symptoms at onset**
Congenital	Birth	CLN10	*CLN10/CTSD*	NGS Enzymatic assay	Microcephaly, dysmorphic features, seizures, hyperkinetic movements;
Infantile	6–18 months	CLN1	*CLN1/PPT1*	NGS enzymatic assay	Decreased head growth, neuro-developmental regression, seizures;
		CLN10	*CLN10/CTSD*	NGS enzymatic assay	Decreased head growth, neuro-developmental regression;
		CLN14	*CLN14/KCDT7*	NGS	Decreased head growth, seizures (myoclonus);
**Late infantile**					
*Classical*	2–4 yrs	CLN2	*TPP1*	NGS enzymatic assay	Seizures, ataxia, visual loss, delayed language development;
*Variant*	2–5 yrs	CLN1	*CLN1/PPT1*	NGS enzymatic assay	Seizures, neuro-developmental regression, behavioral disturbances;
		CLN5	*CLN5*	NGS	Impaired learning and cognition;
		CLN6	*CLN6*	NGS	Seizures, ataxia, delayed language development;
		CLN7	*CLN7/MFSD8*	NGS	Seizures, visual loss, motor and cognitive regression;
		CLN8	*CLN8*	NGS	Seizures, visual loss, motor and cognitive regression;
**Juvenile**					
*Classical*	3–5 yrs	CLN3	*CLN3*	NGS	Visual loss, behavioral problems, cognitive decline
	5–7 yrs	CLN5	*CLN5*	NGS	Motor and cognitive regression, behavioral problems;
	5–7 yrs	CLN1	*CLN1/PPT1*	NGS enzymatic assay	Visual loss, cognitive decline;
*Late*	8–12 yrs	CLN6 CLN10	*CLN6* *CLN10/CTSD*	NGS NGS enzymatic assay	Myoclonic seizures, cognitive decline; ataxia, cognitive decline, visual loss;
	13–16 yrs	CLN12	*ATP13A2*	NGS	Rigidity, hypokinesia

An axial classification system was proposed, including seven axes, to obtain information necessary to better categorize each NCL form, according to the specific items which characterize them (30) and represents a useful diagnostic tool for research purposes.

## General Clinical Issues

### Advances in Genetics

NCL are genetic diseases (13). All childhood and most adult NCLs are inherited as autosomal recessive diseases. There is only one dominantly transmitted adult-onset form, Parry disease, associated with mutations in *CLN4*.

A new age in the NCL history started during the last decade of the 20th century. The first human NCL genes, *CLN1* and *CLN3*, were identified in 1995 by positional cloning (31, 32). The identification of the remaining eleven NCL genes occurred within the following decade, making use also of naturally occurring animal disease models. The pathogenicity of identified mutations was proven by different approaches *in vivo* (eg. using knock-out invertebrate and mammalian models) and/or *in vitro*.

The consequences of the genetic advances are multifaceted. From a clinical perspective it allowed a new gene-based classification and NCL nomenclature (33), provided a powerful tool for diagnosis, and helped clinicians recognize both phenotypic variability and heterogeneity within most NCL genetic disease types (34). The classical phenotypes are generally associated with the most common mutations in each gene, whereas variant forms may arise from “private” mutations. Phenotypic variability can be observed even within some families, which, for example, may lead to differences in survival among siblings. Advances in NCL genetics also gave the opportunity to re-evaluate NCL epidemiology and to recognize the worldwide distribution of this group of diseases (see below Section Epidemiology and Registries). The identification of the NCL genes and their related products helped to begin to identify altered cellular processes and patho-mechanisms leading to cell death and progressive neurodegeneration which characterizes all NCLs. The cross-talk between different cell compartments (eg lysosomes and endoplasmic reticulum, lysosomes and mitochondria) has been described in some NCL forms (35–38) and has helped to shed light on some clinical features which cannot be ascribed to the mutated NCL protein only. Animal and cellular models have been generated to expand our knowledge of the pathology, and so the first disease modifying and therapeutic agents are now becoming available for clinical trials (39). The first such agent was approved for clinical use in 2017 following a pivotal phase I/II clinical trial.

As shown in Table 2, allelic adult-onset variants are observed in several childhood forms (40). In most however, the adult phenotype shows clinical features which are consistent with the classical childhood form and are differentiated only by the age of onset. The well-defined adult onset NCLs are still named by classical eponyms [Kufs A, Kufs B and Parry diseases]. They are associated with pathogenic mutations of *CLN6* [Kufs A disease, (41)] and with three other genes (*CLN4, CLN11, CLN13*) whose mutations give rise exclusively to adult-onset phenotypes (42–45).

**Table 2:** Characterization of adult onset NCL.

**Form**	**MIM**	**Inheritance**	**Chromosome**	**Gene**	**Gene product**	**Onset (decade)**	**Symptoms at onset**
KD-A/CLN6	#204300	AR	15q23	*CLN6*	CLN6: transmembrane protein (ER)	2nd-5th	Seizures, action myoclonus, ataxia, cognitive decline
KD-B/CLN13	#615362	AR	11q13.2	*CLN13/CTSF*	CTSF (soluble protein): lysosomal enzyme	2nd-7th	Seizures, myoclonus, cerebellar tremor, cognitive decline, depression, anxiety
Parry/CLN4	#162350	AD	20q13.33	*CLN4/DNAJC5*	CSPα (soluble protein): cytosol (vesicular membrane)	3rd-5th	Seizures, action myoclonus, visual failure
CLN11	#614706	AR	17q21.31	*CLN11/GRN*	Progranulin (soluble protein)	3rd	Seizures, visual failure
CLN1	#256730	AR	1p34.2	*CLN1/PPT1*	PPT1 (soluble protein): enzyme (lysosome and extra-lysosomal compartments)	3rd-4th	Cognitive decline, psychiatric symptoms
CLN5	#256731	AR	13q22.3	*CLN5*	CLN5: lysosomal membrane (other cell compartments)	6th	Unsteady gait

*KD, Kufs disease; ER, endoplasmic reticulum*.

The identification of childhood NCL genes is also contributing to the identification of potential genetic signatures for neurodegeneration in adulthood. A number of NCL genes share mutations or are allelic with mutations contributing to common adulthood neurodegenerative disease, such as Alzheimer's Disease, Fronto-Temporal Dementia, Parkinson's Disease (46–48). Moreover, lysosomal dysfunction and dysregulated autophagy which are observed in CLN3 and CLN6 disease (49, 50) are also seen in most forms of late-adulthood neurodegeneration (51, 52).

**Figure 2:**
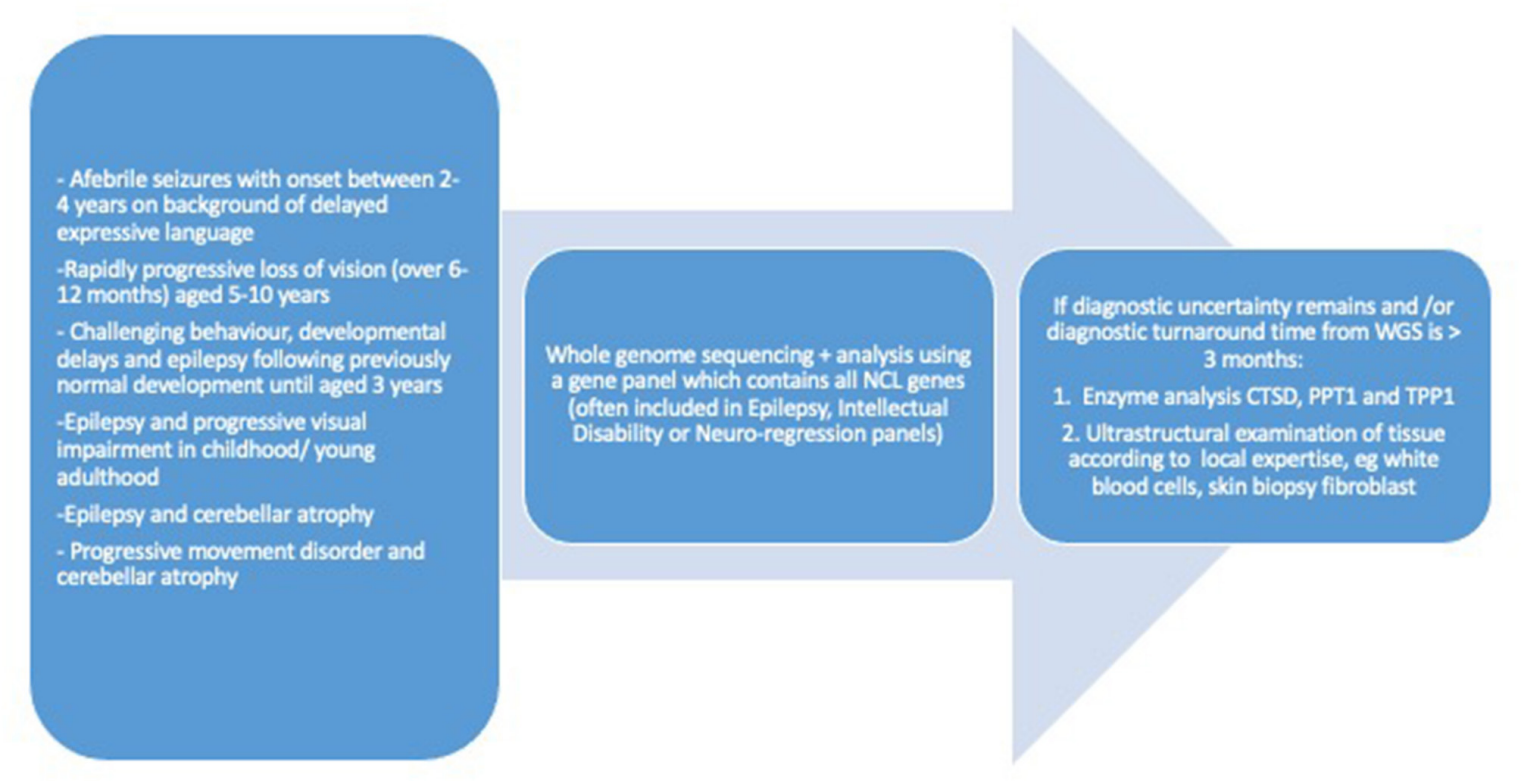
Diagnostic work up for neuronal ceroid lipofuscinosis of childhood onset.

Advances in NCL genetics were accompanied by new opportunities to establish diagnosis at a biochemical level; enzymatic assays became available for four lysosomal enzymes: CTSD, CTSF, PPT1, and TPP1 (53–55). The gold standard for diagnosis is to require genetic confirmation by detecting both disease causing alleles. Therefore, when detection of the common disease causing mutations is not confirmed in the presence of a biochemical impairment, the diagnostic search for changes in cryptic gene regions (introns, untranslated regions, etc.) is extended. The requirement to establish a molecular diagnosis is of a primary importance for families helping to inform prognostic counseling, for antenatal diagnosis and, more recently, to judge suitability for “innovative” treatments where they are available. The recent availability of NGS technology has changed the clinical approach to NCL diagnosis, which relies on a straightforward molecular approach, leaving the neuropathological and ultrastructural investigations to cases which remain unsettled, even after molecular analysis (Figure 2).

### Epidemiology and Registries

NCL are rare diseases which are distributed world-wide. The higher prevalence of selected NCL forms in restricted geographical areas has some historical relevance and may also reflect the early progress in molecular diagnosis in some countries (31, 56, 57). Epidemiological data indicate an incidence of 1–3/100.000 and a prevalence of about 2–4/1.000.000 (58–62). These figures refer to Western countries where access to molecular diagnosis has become standard of care over the last 10–15 years. More detailed epidemiological study is necessary to improve awareness of these diseases, the efficacy of genetic counseling, to plan appropriate services, and to facilitate access to “new” treatments. In Western countries CLN3 disease (juvenile NCL) is the most common form, followed by the classical late infantile form, CLN2 disease which is more frequent in Southern Europe and the Mediterranean region. Ultrarare childhood NCL are CLN10, CLN12, CLN14. A major contribution to the dissemination of knowledge comes from disease registries and databases. The independent International NCL Database (led by Dr A Schulz in Hamburg, Germany; Clinical trials identifier NCT04613089, an extension of former NCL DEM-CHILD patient database) is collecting static and dynamic clinical data of NCL patients from 19 countries from Europe, North and South America and Asia. The UCL based NCL Mutation Database (established in 1998 by prof S.E. Mole; http://ucl.ac.uk/ncl-disease/) lists all published and reported NCL patients as well as mutations.

### The Current Nosography

The impressive amount of data collected over the last two decades has allowed the NCL community to generate a new nomenclature of NCL based on genetic variations (33), followed by comprehensive reviews which have described the clinical features, the cell localization and functions of the mutated gene products, as well as the historical markers which characterize NCL among other progressive neurological diseases with endo-lysosomal storage. For a systematic review of the clinical and diagnostic features of each NCL disease (including the phenotypic heterogeneity), readers are referred to recent publications (1, 63–69). A comprehensive classification of NCL is given in Table 3.

**Table 3:** NCL: genetic, biochemical and ultrastructural features of each form.

**NCL disease**	**MIM**	**Gene locus**	**Mutated gene**	**Gene product**	**Cellular compartment**	**Function**	**Ultrastructural features**
CLN1	#256730	1p34.2	*CLN1/PPT1*	PPT1 (soluble protein)	Lysosomal matrix (acidic ph); extralysosomal vesicles (not acidic pH);	Enzyme (s-acylated protein thioesterase)	GROD
CLN2	#204500	11p15.5	*CLN2/TPP1*	TPP1 (soluble protein)	Lysosomal matrix	Enzyme (serine protease)	CVB (FPP)
CLN3	#204200	16p11.2	*CLN3*	CLN3 (membrane protein)	TMD (late endosomes, lysosomes, presynatic vesicles, axons)	Multiple functions in several cell processes	FPP, vacuoles
CLN4	#162350	20q13.33	*CLN4/DNAJC5*	DNAJC5 (soluble protein)	Cytosol (vesicular membrane)	Co-chaperone (endo/exocytosis)	GROD
CLN5	#256731	13q22.3	*CLN5*	CLN5 (soluble protein)	Lysosomal membrane and matrix, ER, neurites	Endocellular trafficking between cell compartments	Mixed pattern (CVB, FPP, RLP)
CLN6	#601780	15q21-23	*CLN6*	CLN6 (membrane protein)	TMD (ER)	Mediates ER exit of new lysosome enzymes (?)	FPP
CLN7	#610951	4q28.2	*CLN7/MFSD8*	MFSD8 (membrane protein)	TMD (lysosomes, late endosomes; photoreceptor vesicles)	Transporter?	Mixed pattern (CVB, FPP, RLP)
CLN8	#610003 and #600143	8p23.3	*CLN8*	CLN8 (membrane protein)	TMD (ER/ERGIC) (cargo receptor)	Lysosome biogenesis regulator	Mixed pattern (CVB, FPP, RLP)
CLN10	#610127	11p15.4	*CLN10/CTSD*	Cathepsin D (soluble protein)	Lysosomal matrix	Enzyme (aspartyl-endopeptidase)	GROD
CLN11	#614706	17q21.31	*CLN11/GRN*	Progranulin (soluble protein)	Extracellular matrix	Not known	FPP
CLN12		1p36.13	*CLN12/ATP13A2*	CLN12 (membrane protein)	TMD (lysosomes)	Enzyme (P-type ATPase)	FPP
CLN13	#615362	11q13.2	*CLN13/CTSF*	Cathepsin F (soluble protein)	Lysosomal matrix	Enzyme (cysteine protease)	FPP
CLN14	#611725	7q11.21	*CLN14/KCTD7*	Potassium Channel Tetramerization Domain-containing Protein 7 (soluble protein)	TMD (plasma membrane)	Voltage-gated potassium channel complex	Mixed pattern (CVB, FPP, RLP)

*TMD, transmembrane domain; ER, endoplasmic reticulum; ER/ERGIC, endoplasmic reticulum/endoplasmic reticulum-Golgi intermediate compartment; GROD, granular osmiophilic deposits; CVB, curvilinear bodies; FPP, finger print profiles; RLP, rectilinear profiles*.

## Selected Clinical Features

As mentioned in the introduction, the NCLs were grouped as amaurotic idiocies around the end of the 19th century, along with other diseases which were recognized as different disorders later. “Idiocy”, progressive blindness, and seizures, remain the cardinal symptoms of the NCLs. The NCLs are the most common neurodegenerative diseases in childhood, and they are one of the main causes of childhood dementia worldwide. They are progressive with severe physical decline, and an early death. Infantile and late infantile onset NCLs show the most rapid rate of disease progression. Most patients are bedridden by their second decade, and death occurs during the late second-early third decade. A less rapid course is seen in patients affected with CLN3 disease. Staging systems based on severity scores of selected clinical manifestations have been developed to monitor the disease evolution quantitatively. These tools are helpful in describing the natural history of a disease and are of particular relevance when the efficacy of “new” treatments is tested (70–72).

Cognitive decline, ataxia, amaurosis, and early seizures are the clinical manifestations at onset in most cases. They become evident sequentially over a short time frame (6–24 months) in infantile and late-infantile NCLs; spasticity follows leading to loss of motor function, dependence on carers for all activities of daily living within 5–8 years of symptom onset.

In this review we will discuss the clinical symptoms which characterize the NCLs in more detail, and are shared between the forms, focusing on their temporal evolution at onset and during the early stages of the disease course. Seizures, motor deterioration (including ataxia, movement disorders, spasticity), cognitive decline, progressive blindness, and behavioral problems are the major clinical features of infancy and childhood onset NCLs. The identification of the clinical markers and their relationship with patients' age at onset may help to drive diagnostic procedures toward a specific NCL type, which can be confirmed molecularly. Such an approach reflects the clinical experience of the authors in the field of childhood neurodegeneration.

### Epilepsy and Seizures

Epilepsy is common to almost all NCL forms. Several seizure types are seen in the NCLs. The severity of epilepsy (in terms of age of onset, seizure semiology, seizure burden and response to anti-seizure medications) is not shared uniformly. Seizures tend to start earlier and semiology is more varied in infantile and late-infantile onset NCLs. With time seizures tend to become less frequent, except for myoclonus (either spontaneous or evoked) which remains the only paroxysmal manifestation in the late stages of the disease. The background EEG progressively deteriorates leading to a nearly isoelectric pattern, as an expression of progressive cortical atrophy and degeneration of the “generators” of cortical electric activity.

Frequent perinatal *convulsions* (or even status epilepticus) associated with severe cortical and cerebellar atrophy are observed in the ultrarare congenital NCL, which leads to death within the first weeks of life and are usually associated with mutations in *CTSD* (73).

*Generalized seizures, including myoclonus*, starting 2 to 6 months after the earliest clinical manifestations, characterize infantile onset NCL (infantile CLN1 and CLN10 diseases). Before the onset of clinical seizures, EEG abnormalities can be detected, which evolve over time into a characteristic pattern, the “vanishing EEG”, observed also in the late infantile variant of CLN1 disease (74–76).

*Generalized motor seizures, absences, and myoclonus (including negative myoclonus*) are the main seizure types observed in classical CLN2 disease. They are also seen in CLN6, CLN7 and CLN8 late infantile variants. In these forms seizures can be present at disease onset, or appear within 1–2 years. A characteristic EEG feature is the paroxysmal spike-wave response, which is evoked by low-frequency (1–3 Hz) intermittent photic stimulation which is observed in CLN2 and CLN6 (late infantile variant) patients during the early stages of the disease (77, 78).

A prevalent *myoclonic epilepsy with progressive features* characterizes the late juvenile (teen age) onset *CLN6 disease* and its allelic adult Kufs A disease. Both forms present with progressive myoclonic epilepsy at onset gradually increasing in frequency and intensity, and less frequent bilateral tonic-clonic seizures. A low-frequency (1–5 Hz) photo-paroxysmal response is present in the vast majority of cases (79). The disease has a slowly progressive course, and patients become wheel-chair bound within few years due to the relentless myoclonus and progressive ataxia (80). Progressive myoclonus epilepsy associated with rapidly progressive dementia is also the clinical hallmark of the dominantly inherited NCL (Parry disease) (42).

*Bilateral tonic-clonic* seizures (even with focal onset) and *absences* are the most frequent seizure type in CLN3 disease, rarely occurring as presenting symptoms; myoclonus is less frequent than in other NCLs (81, 82).

### Major Neurological Symptoms

#### Motor Deterioration

Ataxia, weakness, loss of acquired motor abilities and spasticity are the most common motor symptoms in NCL patients. The consequence is the loss of motor autonomy and increasing dependence on caregivers. Signs and symptoms of motor impairment become evident in children who had normal motor development previously.

*Ataxia* is the most frequent clinical symptom of CLN2, CLN7, CLN8 disease and of the rarer late-infantile variant of CLN1 disease whose age of onset is between 2 and 5 years. It is also evident in CLN6 disease but onset is 1–2 years later. Ataxia in the NCLs is secondary to spino-cerebellar involvement. Purkinje cells and neurons of the deep cerebellar nuclei are severely affected and undergo early death. Cerebellar atrophy is an early neuro-radiological sign, preceding the enlargement of cortical sulci and of the lateral ventricles. Sometimes the term “ataxia” is incorrect, and the transient spells of motor instability which a child shows should be considered as clinical expression of “negative myoclonus”, which is commonly present in these forms.

*Motor weakness* affecting limbs, eye muscles (strabismus) and deglutitory muscles (dysphagia) is likely to occur because of impaired cortico-descending connectivity due to the involvement of motor cortex and the neurons of motor nuclei. The progressive degeneration of motor pathways is followed by *spasticity* which represents further disease progression. Changes in gait phenotypes in different forms of Batten disease has been recently considered as a “marker of disease progression” (83).

The progressive involvement of cortical neurons also leads to a progressive *loss of motor skills, motor initiative, motor planning and strategies*, which represent the “cognitive foundation” of motor function. This same process also leads to dementia. Declining motor function is observed in all NCL forms, leading to loss of independent ambulation, subsequent loss of all voluntary movement and posture control, the rate of decline dependent on the NCL type. Children with the infantile and late infantile NCL types become wheel-chair bound at 6–12 years of age (according to the severity of the form) before becoming “bed-ridden” (early teens in late infantile forms, early-mid second decade in CLN3 patients).

*Extrapyramidal symptoms*, such as tremor and rigidity, are known to occur in NCL, being particularly relevant in CLN3 disease. They are responsive to available treatments, such as anti-cholinergic drugs, Dopamine. Dystonia and chorea can be recognized at different stages of NCL disease course (84, 85). The reasons why basal ganglia structures and/or neuronal connections underlying these clinical manifestations are relatively spared in NCLs is a matter which requires further investigation.

#### Cognitive Decline and Impaired Language Development

Cognitive decline is hallmark of the NCLs. In younger children the loss of cognitive abilities related to school-learning occurs rapidly, whereas the relatively slow pace of disease evolution observed in “juvenile” patients allows them often to attend mainstream school whilst continuing to learn new facts and skills into their teenage years. From this perspective two major issues should be considered, which further differentiate early onset NCL from juvenile forms.

Cognitive decline occurs as a two-phase problem. Early in the disease, children's developmental trajectory slows, they acquire new learning more slowly than their peers but often remain within the normal range for some time before professionals become concerned. They go on to plateau and then begin to lose the cognitive competences they have acquired during the earliest months and/or years of their life. This is often the time at which diagnostic investigations are triggered. In CLN2 disease however many children show a delayed acquisition of cognitive skills at a very early stage and their profile is characteristically uneven with delays more evident in the domain of expressive language compared with motor skills. This has been reported in recent years and is of huge importance for driving early diagnosis. Nickel et al. (86) reported that about 40% of a cohort of CLN2 patients presented early with delayed language acquisition. It raises the potential to consider language delay as an early warning sign, leading to careful clinical examination of such children, in order to detect any additional concerning features which would justify a deeper diagnostic workup including genetic screening. The atypical early developmental profiles of affected children may also give some clues about the molecular basis of language development, and the role that cellular pathways involved in the development of this complex function may play. There are no studies available for NCL late infantile variants, but it is tempting to hypothesize similar findings in some NCL types at least, e.g. CLN7 and CLN8 disease. Delayed language development in CLN2 disease children does not seem to be related to any specific CLN2 genotype.

Different clinical issues are related to the decline of cognition in CLN3 disease, which can be considered as the prototype of amaurotic idiocy. The rate of disease progression is commonly slow and children can attend school, with support for the intellectual, visual and sometimes behavioral difficulties. There is no evidence to date of an uneven developmental profile early in life and before the onset of visual impairment. The scores of the neuropsychological function tend to diverge from typically-developing peers, reflecting the failure to achieve the expected achievements, but a marked drop in their cognitive abilities occurs during mid-adolescence, at around the same time as rapid neurological decline (87).

#### Behavioral Problems

Behavioral problems at onset characterize CLN3 and CLN5 diseases, and to a less extent atypical CLN2 disease, three NCLs where first symptoms occur in school age children. There are well described and differentiated disease natural history, and which are caused by mutations in three different nuclear genes whose products have different functions and are located in different cell compartments.

CLN3 disease is the most prevalent NCL in Northern Europe and USA. It has a juvenile (school age) onset with *visual impairment and behavioral problems*, followed by cognitive decline. Motor impairment and epilepsy occur later. Behavioral problems include anxiety, depressed mood, bursts of aggressive behavior and psychotic manifestations. These symptoms tend to remain stable or even worsen during the early years after disease onset, and then become less significant as the disease advances and functional and cognitive abilities are lost. Behavioral problems represent a major challenge to the quality of life for those patients (and their families and carers) where a slow disease course and longer survival is expected (87, 88), and seems also to be unrelated to the genotype (89). The disease evolution is much slower than in other forms, and death may occur in the fourth or even sixth decade, unless cardiac involvement results in premature death (see below). There is a relevant phenotypic homogeneity (possibly related to a common mutation which is observed in the vast majority of cases). Some gender differences have been reported, the female patients presenting a more severe clinical course (90).

*Behavioral manifestations and impaired language* are the early clinical manifestations of a late infantile “variant”, of pre-school age onset, CLN5 disease. Seizures and visual impairment occur 3–4 years after disease onset, followed by progressive motor impairment with spasticity with loss of ambulation by 11–12 years of age. The predicted survival is about 15–20 years after disease onset. In this disease there is some evidence that phenotypic variation (as far as the rapidity of neurological decline and survival) is related to the severity of mutations (91, 92).

*Behavioral disorders (*along with *seizures and, language abnormalities*) at onset were also reported in a large South American cohort of children with *atypical CLN2 disease* (93).

#### Blindness

Progressive visual impairment is one of the classical symptoms of NCL of childhood onset. It affects all forms, and in the large majority of cases it occurs as one of the early clinical signs. Retinal structures, visual pathways and visual cortices are affected. Both ganglionic neurons and receptor cells (cones and rods) are involved. Impaired retinal response is visualized by ERG. Optical Computerized Tomography allows monitoring of the progressive retinal degeneration. Most published evidence relates to CLN3 and CLN2 diseases (94, 95), and to less extent to CLN1 disease (96). A spectrum of retinal disturbances was described in CLN7 disease and related to the severity of mutations (97). Whether maculopathy precedes cognitive impairment in CLN3 disease, or if both visual impairment and mild cognitive failure occur at around the same time (98) is still the subject of discussion. Biallelic variants of the MFSD8 gene may be associated with isolated juvenile maculopathy, evolving into a slowly progressive encephalopathy with protracted course (99). Conversely, late visual impairment, or even long lasting preservation of visual function can occur in CLN5 and CLN6 diseases (92, 100). Notably, in the allelic Kufs A disease the retina is not affected and vision is normal. Recently, patients affected with isolated retinal degeneration (including retinitis pigmentosa) have been identified harboring biallelic CLN3 and CLN10 pathogenic variants (101, 102).

Therapeutic trials are in progress aiming to prevent retinal degeneration in animal models using intravitreal gene or enzyme therapy (103, 104). These approaches represent powerful therapeutic systems suitable for translation into NCL patients. A major question arises regarding the contribution of central nervous system pathology to functional vision, which is unlikely to be corrected by a retinal only approach.

#### Sleep Disturbances

Sleep disturbances are common in all NCL types. There are few studies on this issue (105–107) but the importance of sleep management has been outlined in two recent consensus papers (108, 109). Poor sleep quality and sleep pattern disturbance will impact adversely on the quality of life of affected children and their families directly and indirectly through worsening of seizure control and behavior. Supportive measures (such as respite care) and behavioral strategies (such as sleep hygiene) are necessary given the lack of consistent benefit from commonly used medical interventions, such as Melatonin (110). Anti-seizure medications, often prescribed, may help falling asleep, but side effects (e.g. morning drowsiness) impair the restorative function of sleep.

### Atypical Cases

It has been known for several years that phenotypic heterogeneity is a feature of genetic diseases (including NCL), and some patients were considered as atypical cases. The origin of clinical variation is commonly ascribed to the genetic background of patients as well to the severity of the mutations. Recently polymorphisms or mutations of unrelated genes have been considered as modifiers of gene expression, and the interactions between mutated genes and modifiers may lead to clinical variations and to the observed phenotypic heterogeneity. In addition non-genetic confounding factors may also affect clinical phenotype.

An “atypical case” has been described in a child affected with a congenital form of CLN5 disease (which usually presents with a late infantile onset, with relatively slow disease progression after onset): he was a compound heterozygote in *CLN5* and was also carrying an incompletely penetrant variant in *POLG1*, a nuclear gene coding for mitochondrial polymerase (111). No evidence was provided of how the co-inherited *POLG1* variant may have enhanced the putative pathogenetic effect of the *CLN5* mutations. However, the interactions between lysosomal and mitochondrial compartments may be implicated, further affecting the physiology and viability of cells hampered by the presence of pathogenic *CLN5* mutations. Another atypical case is related to *CTSD*, commonly associated with congenital or late infantile NCL (CLN10). In a patient with juvenile ataxia associated pigmentary retinopathy and cognitive decline, the evidence of granular osmiophilic deposits (an ultrastructural marker of CLN1, CLN4 and CLN10 disease) in angular atrophic fibers following a muscle biopsy led to the final diagnosis (29). Unusual phenotypes associated with mutations in *CLN2*/*TPP1* with residual leukocyte enzymatic activity raise the issue of the phenotypic heterogeneity, regardless the severity of mutations. A prevailing spino-cerebellar involvement was detected in an adult lady who was suffering from ataxia since adolescence. She was carrying a missense and a splice site variant in *CLN2*/*TPP1* (112). A juvenile onset, progressive and protracted form of cognitive decline with myoclonus, dystonia, bradykinesia and ataxia was reported in three siblings and associated with compound heterozygosity in *CLN2/TPP1*, leading to stop codon formation and an aminoacid substitution (113).

It is evident from these “atypical cases”, that the detection of a mutated gene represents the starting point to explore the complex mechanisms which ultimately lead to the phenotypic expression, taking into account the unique genetic background which each of us has inherited. Improving our knowledge in this field of “personalized medicine” will help to prevent unexpected and potentially serious adverse events, which might arise when using conventional and novel replacement therapies.

### Cardiac Involvement

NCL are considered as LSD. However, they do not show the characteristic multi-organ involvement typical of many LSDs (114), and they predominantly affect the central nervous system. Progressive cardiac involvement is observed in some patients affected with CLN3 disease with repolarization disturbances, ventricular hypertrophy, and sinus node dysfunction (115). Cardiac pace-maker implantation has been used in a small number of patients to improve symptoms of syncopy and extreme bradycardia but is not in widespread use. Twenty four hour ECG recordings have been recommended for annual surveillance in young people age 16–18 years and older. Degenerative changes of the myocardial wall along the deposition of lipopigment and cytosomes can be detected histologically (116).

Heart involvement has also been reported in CLN2 disease [progressive conduction defect; (117)] and hypertrophic cardiomyopathy was described in patients with CLN10 disease (29, 76).

### Care and Survival

Reports on life expectancy and mortality in NCL are scarce (118). This issue is assuming particular relevance in natural history studies when assessing the efficacy of “orphan drugs”.

The ultra-rare congenital form of NCL has the most dramatic course, the very few reported cases having died within two weeks of birth (119). Ongoing studies confirm that infantile-onset NCL (due to mutated CLN1 and CLN10 genes) and classical CLN2 disease have the next most rapidly severe course and shortest survival. Variability within each form does not allow accurate predictions of life expectancy in individual patients According to the Italian NCL database CLNet, death in the majority of CLN2 patients has been around nine years of age, but prolonged survival is reported in a number of patients affected with the classical form, up to the early third decade, regardless the predicted severity of the mutations they were carrying. Intra-familial variability is also reported. Little is known for the remaining NCLs with infantile and late infantile onset. As mentioned before, the longest survival (up to 4th-6th decade) is observed in CLN3 patients.

Over the last two decades overall longer survival has been observed in patients of many NCL forms, regardless the severity of mutations. Such findings may be ascribed in large part to the improved availability of supportive tools for home care which guarantee appropriate feeding (e.g. percutaneous gastrostomy), respiratory support, as well as the improved quality of skin and general care (e.g. prevention of bed sores), the availability of new anti-epileptic drugs, and the overall improved awareness of caregivers to provide care in order to enhance the length and quality of life of those affected (120, 121).

## The Research Contribution to Knowledge

The NCLs are progressive neurodegenerative diseases associated with endolysosomal storage. No cure is available, and major efforts aim to provide the best care to patients. Recently, disease modifying agents have become available or are under investigation for some NCLs (122), by replacing a missing enzyme, the mutated gene, or by reducing the substrate involved in abnormal storage formation. Such approaches reflect the ongoing lack of understanding of the basic disease mechanisms specific to each form, as well as of the shared pathways which characterize the whole group of NCL diseases, despite extensive research efforts.

Experimental paradigms and innovative methodologies have provided major breakthroughs over the last two decades on the knowledge of pathological mechanisms underlying the NCLs (123). In this paragraph we summarize the most relevant data resulting from the current research under different experimental settings, which may contribute to better understanding the rationale of the research toward appropriate strategies to generate new, safe and effective treatments.

Recent studies of outcome on a relatively large population of CLN2 patients are revealing a meaningful slowing down of the rate of disease progression, following the availability of the first “new treatment” (recombinant pTPP1 delivered intrathecally into the ventricular system). Great expectations have risen from the recent announcements of forthcoming treatments whose aim is to both increase the survival and ameliorate the quality of life of the affected children.

### Experimental Models

#### Naturally Occurring Animal Models

Several naturally occurring animal models of NCLs have been discovered and investigated, including non-human primates, ovines, felines, dogs and rodents. The use of relatively large mammals, with convoluted gyral brains represents a powerful tool to examine disease evolution over time (from the early stages until death) and monitor brain pathology using *in vivo* techniques (head imaging, neurophysiology etc) which are currently in clinical use (124–126). In addition, multiple techniques can be utilized to investigate post-mortem brains (and eye) from different perspectives. Along with classical descriptive neuropathology, methodologies are now available which can help to disentangle the complex network of interacting events accompanying the disruptive effects of the intra-neuronal storage in complex mammalian brains. They include the use of immuno-histochemical probes, which allow the detection of selected neuronal and glial populations, to evaluate the distribution and topography of the storage, to check the expression of markers related to pathological processes (such as autophagy), and more recently omics techniques (see below) which allow exploration of expression at the metabolic, protein or even nucleic acid levels of selected biochemical pathways and/or genetic markers (127).

Moreover, these animals are essential in the evaluation of “new” treatments, testing safety, efficacy and putative effects, before progressing to human clinical trials [(39, 128); see Section Pathology and Pathogenetic Mechanisms]. In Supplementary Table 1 some examples of selected animal models contributing to our understanding of disease mechanisms (and treatment) related to specific human NCL forms are listed.

#### Engineered Animal Models

Most of the present knowledge of the patho-mechanisms of NCL comes from experimental studies using mouse models which were modified in syntenic NCL genes. A number of labs in Finland, UK, USA, and Germany have generated mouse models of NCL diseases (*CLN1, CLN2, CLN3, CLN5, CLN6, CLN7, CLN8, CLN10*). Gene targeting technology has allowed selected mutations within a gene to be engineered, offering an experimental background more alike to that in human disease. Generally large numbers of experimental animals can be created, and survival is long enough for a high number of experiments to be carried out. This is the major advantage as compared to spontaneously occurring large mammals, where the number of animals carrying homozygous mutations for a selected gene is relatively low and experiments need to gather as much information as possible from each animal. Advances in technology allow engineered mice to be investigated according to the same methodologies described for large mammalian brains (for example imaging and neurophysiological studies).

#### Simple Cellular Systems

The availability of miniaturized technology has allowed the use of high throughput systems including invertebrate such as Zebrafish, Drosophila, the social amoeba Dictyostelium, yeast, *in vitro* cell systems, such as SH-SY5Y Neuroblastoma cells, and from human patient fibroblasts (obtained from skin biopsies). The use of these simple cellular models make them suitable to address selected questions and obtain results from targeted experiments which are easily repeatable and provide high statistical power (129).

#### Human Fibroblasts and Derived Cells

A major advantage of using human fibroblasts is the ability to transform them into neuronal cells through a complex process *in vitro* of diversion from the primary cell lineage, regression of the cells into induced pluripotent stem cells (iPSCs) and transformation into neurons using *ad-hoc* enriched media. Such studies are already in progress: neuronal cells, derived from fibroblasts of CLN5 patients, showed features consistent with a NCL phenotype (130), and transformed neurons, derived from fibroblasts of CLN2 and CLN3 patients, showed impaired activities of the lysosomal-mediated pathways (131).

A major limitation of this technology, however, is that fibroblasts carry the genetic background of the affected patient, so that direct comparisons between the phenotypic variations of a specific mutation among cell lines from different patients and inferences about the functional implications of specific mutations are challenging. This difficulty can be addressed by combining the CRISPR/Cas9 genome editing technology to generate targeted mutations in mammals as well as in healthy human iPSCs or in human embryonic stem cells (132, 133), without the bias of the potential effect of the mutated gene on the native genetic background.

A further development in the *in vitro* technology, using human cells to investigate the functional effects of individual mutations, is the generation from iPSCs of cerebral organoids (134). Such an approach allows investigation of the effects of a mutated gene not only on the mature cells, but also to evaluate its putative involvement in general neurodevelopmental mechanisms. The available technology makes it possible to test hypotheses multi-Omically, and therefore obtain information about the main pathways and functions modified by the mutated gene during the mini-brain development (135).

### The Omics Approach: Aspects and Significance

Omics approaches represent recently developed technologies which provide high-throughput data related to the genome (DNA) the transcriptome (RNA) the proteome (protein), and the metabolome (metabolic products). The integrated study of data derived from Omics investigations represents the foundation of system biology, and it has been applied to investigate disease mechanisms, particularly the identification of affected biological pathways, which may become therapeutic targets as well as potential biomarkers (127). The new knowledge acquired from these methodologies is predicted to contribute to significant advances in the field of NCL research. The use of Omics technologies to NCLs is relatively recent; several issues have been addressed in both human and experimental animal models of several NCL forms. Major studies performed on tissues from patients affected with different NCL forms are summarized in (Supplementary Table 2).

### Pathology and Pathogenetic Mechanisms

Neuronal death is the disease hallmark shared among all NCL forms: it affects CNS neurons, ganglionic neuronal cells of the retina and even ganglionic neurons of intramural ganglia of the bowel wall. All gray regions of the brain are affected by neuronal death, showing however differential patterns in the topography of neuronal loss, the rate of progression and the secondary involvement of the white matter. Selective neurodegeneration, targeting specific regions and particular cell populations, can be observed during the early stages of the disease, and the patterns of disease evolution can be monitored by neuroimaging studies (136–140). Whether these features reflect the genetic heterogeneity of the NCL is a matter to be investigated further. As clearly indicated by the clinical symptoms at onset and by the patterns of disease progression, cerebral and cerebellar cortices are the most affected brain regions in humans. Selective hippocampal pathology has also been described in different NCL (141). In some forms, particularly in CLN2 disease, the rate of atrophy of the cerebellar cortex is faster than observed in the telencephalic cortex. Less marked is the involvement of the basal ganglia in the early stages of the disease, but eventually generalized atrophy of all gray structures is observed in all childhood NCLs. The neuronal loss leads also to progressive atrophy of the centra semi-ovale, due to the lack of axonal projections from and to the cortex, which is accompanied by progressive enlargement of the ventricular system. Traditional human neuropathological studies have shown that neurons of the spinal cord are also affected in many NCL forms (1, 27, 142). These findings emphasize further the generalized susceptibility of all neuronal cells to this condition.

#### Cell Pathology

Most information concerning the selective involvement of gray structures and the neuronal loss in the NCL brain come from elegant studies carried out using mouse models, which allowed descriptions of the temporal evolution of neuronal loss, established the role of both astroglia and microglia in the brain pathology (143), dissected out the most vulnerable cell compartments involved in neuronal degeneration and proposed hypotheses regarding the cellular mechanisms and pathways underlying the progressive neuronal loss seen in the NCLs.

##### Topography of the Lesions

A gradient of neuronal degeneration is commonly observed, affecting the cortex, the cerebellum and the thalami. Thalamic gliosis seems to precede the onset of cortical involvement (neuronal loss) in both CLN1 and CLN10 mouse models (144) and early thalamic involvement is considered as a radiological marker of LSD (145). A reverse pattern of degeneration is observed in CLN5-/- mice (146). Recently, neuronal loss has also been described in in a mouse model of the disease in the spinal cord, preceding the neuronal loss of the remaining brain regions (147).

Such gradients of evolution are less evident in human post-mortem studies, in which all the brain structures are severely affected in a similar manner: neuropathological examinations are performed several years after the disease onset.

##### Selected Involvement of Neuronal Cell Structures

Neuronal death represents the end point of a complex process to which several processes contribute. However, some groups of neuronal cells seem to be more vulnerable to the pathological condition, as shown by some elegant experimental studies.

Axons are affected: early axonal breakdown was shown in the CLN1 KO mouse model (144); impaired elongation and branching, giving origin to a stunted growth was observed in vitro, in a neuronal-like cell system, overexpressing CLN1 gene (148).

Several aspects of synaptic pathology were also reported. Loss of synaptic proteins was reported in both mouse (CLN1) and ovine (CLN5) models of diseases (144, 149), as well as impaired synaptic vesicles recycling (150). Impaired NMDA-R development was described by Koster et al. (151). Recently, a reduction of functional voltage-gated Calcium channel, in differentiated SH-SY5Y cells, overexpressing CLN1/PPT1 gene was reported (152).

Results from different experimental techniques provide evidence that neuronal connectivity is affected in several NCL models. Impaired cellular function due to the distorted topology of the neuronal cells because of the intra-lysosomal storage is amplified by the impaired neuron-to-neuron communication, strongly contributing to neuronal dysfunction and subsequently cell death.

#### Pathogenetic Mechanisms

The molecular mechanisms leading to endo-lysosomal storage formation have not so far been fully elucidated. It should be noted that a primary defect of lysosomal proteolytic activity is present in only four NCL forms, whereas in the remaining NCLs, storage accumulation is associated with impaired cellular degradation of large molecules by means of a complex pathway, in which lysosomal hydrolytic enzymes are a major, but not exclusive, component. Likewise, which mechanisms link the formation of the endo-lysosomal storage and death of the neuronal cells remains unknown (38).

##### Apoptosis

*Apoptosis* was considered to be the main mechanism leading to cell death following the detection of targeted markers in canine, ovine and human brains and photoreceptors (153, 154). Subsequently, the role of autophagy was investigated in the NCLs, possibly because of the evidence of a temporal link between autophagy and apoptosis (155). Activation of unfolded protein response and apoptosis was shown in the brain of an early mouse model of CLN1 disease (156). The presence of apoptotic cells and expression of apoptosis markers along with lysosomal dysfunction and autophagic stress were also detected in the CNS of a mouse model of Cathepsin D deficiency (157).

##### Autophagy

The evidence of lysosomal accumulation of subunit c of the mitochondrial ATPase F0 complex suggested this occurred secondarily to impaired degradation pathways. Cao et al. (49) showed that *autophagy* was severely affected in a mouse model of CLN3 disease. Moreover, the detection of cell death following inhibition of autophagy suggested that activated autophagy represents a pro-survival response of the cell to the disease process.

Recently impaired autophagy was demonstrated to occur in different experimental models of a number of NCL forms and supported the increasing evidence that dysregulated autophagy is commonly detected in the LSDs (158).

Fibroblasts of patients mutated in *KCTD7* (associated with NCL14) had impaired autophagy (159). Impaired autophagy was also detected in a KO mouse model for CLN1 disease, which is associated to mutated palmitoyl-protein thioesterase-1 (PPT1), a lysosomal enzyme that catalyzes the deacylation of S-palmitoylated proteins. Such a defect was associated with impaired palmitoylation of a protein (Rab7), crucial for autophagosome-lysosome fusion, and therefore leading to impaired degradative function along the autophagic pathway (160).

A role for *CLN5* in dysregulating autophagy was recently suggested by studies in patient fibroblasts and *CLN5*-deficient HeLa cells which showed increased autophagy flux (161). Likewise aberrant development was reported in Dictyostelium deleted in *cln5*, homologous to human CLN5 gene in which autophagy seems to play a regulatory role in terminal differentiation of the amoeba (162).

Interestingly, none of the recently described four experimental conditions, related to mutations in genes associated with different NCL, and associated with impaired autophagy, are associated with genes belonging to the autophagy degradation pathway. That implies that inhibited and/or dysregulated autophagy is the endpoint of a complex network of molecular interactions occurring in NCL neurons, whose outcome is the death of neuronal cells.

##### Oxidative Stress and the Mitochondrial Machinery

Along with investigations directed to the basic mechanism of cell death (such as apoptosis and autophagy), a large amount of data has been obtained over the last two decades from studies aimed at understanding more about the cell physiology in NCL, and other mechanisms which might hamper cell viability and therefore contribute to the cell death process.

Oxidative stress was investigated early, as it was also suggested by the clinical anecdotal observations of transient, worsening of the general wellbeing of affected children under energy-requiring circumstances (e.g. fever, general anesthesia).

Impaired activities of OxPhos enzymes were reported in CLN1 fibroblasts and its putative role on triggering apoptotic cascade was well established (163, 164). Moreover, structural abnormalities of the mitochondrial reticulum as well as abnormal ROS production were described in human fibroblasts (165, 166). More recently, a quantitative proteomic study showed impaired mitochondrial function in different human cells (knocked out in *CLN5*) models and in Cln5–/– mouse cerebral cortex. Impaired autophagy machinery coupled with mitophagy activation processes were observed linking the CLN5 protein to the process of neuronal death (37).

The involvement of the mitochondrial machinery is not surprising in neurodegeneration (as either primary or secondary event) because of the high dependance of neurons on energy supplied by the oxidative metabolism. These findings, however, reinforce the recent evidence of cross-talk between different cell compartments (including lysosomes and mitochondria, as well as endoplasmic reticulum) which seem to be affected differentially in the NCLs. Greater understanding of the molecular relationships of such intracellular “dialogues” might provide clues toward targeted treatments for specific NCL forms (35).

##### Immunomodulation and the Inflammatory Response

As in several neurodegenerative disorders (including Alzheimer's disease), inflammatory changes of the neuropil are significant in PPT-1 deficient mice, including the increased production of pro-cytokines, recruitment of inflammatory cells, microglia activation (167–169). The inflammatory response has therefore been considered a powerful amplifier of the NCL disease pathology. Such findings led to several trials using selected immunomodulatory drugs, which alleviated neurological symptoms in the affected animals, but were more effective if applied before the onset of symptomatology (170). The same rationale lead to a clinical trial for juvenile NCL patients (CLN3 disease), using mychophenolate, an immunosuppressant used off label for autoimmune neurological conditions. The drug was tolerated, but there is no evidence so far for a positive effect on clinical disease progression (171).

## Treatment: The State of Art

### General Issues

The medical management of children and young adults affected by one of the NCLs continues to be mainly symptomatic, delivered through a multidisciplinary and multiagency approach, working closely with family members and carers. Medical care should follow internationally agreed standards and guidelines for individual symptoms and organ systems (for example epilepsy, respiratory, orthopedic and gut) and be delivered in line with the holistic values of palliative care. These approaches with particular reference to the NCLs have been described in a number of publications in recent years (108, 109, 120). Experts in rare diseases should be cognisant of advances in these areas or at least be able to signpost families and carers to appropriate expertise. Some advances have been mentioned in previous sections of this review. In particular, advances in management of chest symptoms in children with complex neurodisability and dependence, including for example use of cough assist devices and non-invasive ventilation, together with cardiac pacing suggest sick sinus syndrome. Newer tone management and anti-seizure treatment modalities (including drugs, dietary therapies and stimulation techniques) should be considered and discussed openly when considering the goals of care and potential medical interventions with families.

### Epilepsy and Tone/Movement Disorder Management

Perhaps the most troubling clinical symptoms are seizures throughout the disease course and tone/movement disorder management in the later stages.

These are widely reported by parents to be the most worrying and by professionals as the most challenging. Many NCL forms of early childhood onset are characterized by a progressive myoclonic epilepsy syndrome whereas in CLN3 disease with juvenile onset generalized motor and absence seizures predominate. Clinical experience suggests that anti-seizure medications considered best for generalized genetic epilepsies are most effective in these disorders whereas those which are known to exacerbate myoclonus are best avoided. The most commonly used anti-seizure medications are valproate, levetiracetam and the benzodiazepines in varying combinations. Carbamazepine is avoided. Lamotrigine is reported to be helpful in combination with valproate in the later stages of CLN2 disease and is very effective at high doses in older children and young adults with CLN3 disease. Experience of international clinical experts varies but there is consensus that medications for epilepsy are used to alleviate seizure burden rather than with the goal of complete seizure freedom and that drug combinations are usually necessary. In some patients the seizure burden is less at the late stages of the disease and medication can be reduced. Ketogenic diet is not contraindicated, is often very well tolerated and may also be helpful.

Movement disorders are increasingly recognized in the NCLs. In several NCL forms with onset in the preschool or early school years (CLN2, CLN5, CLN6, CLN7, CLN8) a choreo-athetosis or mixed movement disorder with dystonia becomes evident a few years after symptom onset. Determining which involuntary movements are ictal and which are movement disorder can be challenging and EEG video telemetry with event capture can be very helpful. The movement disorder progresses to spasticity in the late stages of all NCL forms, complicated by spinal scoliosis and joint contractures. Baclofen, trihexyphenidyl, clonidine and botulinum toxin injections are used extensively and in combination.

Gabapentin has proven very useful for irritability and distress in the rapidly deteriorating phase of CLN2 disease.

### Orphan Drugs and New Treatments

We are beginning to recognize new clinical phenotypes for the few NCL types now amenable to disease modifying therapies such as enzyme replacement and gene therapy. These treatment approaches are new and the full range of implications on symptoms and quality of life as well as survival and longevity is yet to be established. It may well be that there should be a shift from a mainly palliative approach for symptom control to more aggressive intervention with the expectation of complete symptom control (for example seizure freedom) in treated individuals. Close working with the patients themselves, families, caregivers and family representative organizations will be crucial as we go forward to define what standard care should look like.

In LSD and more recently in NCL therapeutic strategies have emerged with the aim of preventing abnormal storage formation and/or to deplete the abnormal endo-lysosomal accumulation, with the ultimate goal to reduce and/or stop the disease progression (122, 172). Several challenges must be overcome, for example the route of drug delivery to the CNS, safety, outcomes and how best to measure efficacy, and above all the still incomplete knowledge of the patho-mechanisms underlying each NCL form. This is still the age of a “gross” therapeutic approach, using replacement therapies (the mutated gene or the missing enzyme), whereas more classical pharmacological treatments targeting crucial steps of the important metabolic pathways still lag behind. It is likely that a combined approach will be necessary to achieve the good therapeutic outcomes patients, families and professionals would like to see.

#### Complementation Therapy

*Complementation therapies* (either as gene replacement therapy or enzyme replacement therapy, ERT) are becoming available for some NCL forms (Table 4). ERT became available as part of a clinical trial almost a decade ago for CLN2 disease. Following the decision of the regulatory agencies (FDA and EMA), the recombinant lysosomal enzyme, cerliponase alpha, replacing the ineffective gene product has been commercially available since 2017 (Brineura©). The enzyme is administered directly to the CNS via an intra cerebro-ventricular catheter every 2 weeks. The safety of this approach is well documented by the most expert group with this procedure (173). As for its efficacy there is good evidence that disease progression is slowed down over a 4 year period, as compared with historical untreated controls (174, 175). Likewise, slowed disease progression was observed in a small cohort of CLN2 children (assessed for a shorter period of time), who received intracerebral injection of adenovirus expressing *CLN2* (176). Even more interesting results were reported in a very small group of pre-school children who underwent treatment before or a few months after clinical onset of the disease, with delayed onset of disease symptoms as well as maintenance of early cognitive skills (177, 178).

**Table 4:** Selected therapeutic trials (completed and on-going).

**Disease**	**Therapy**	**Study**	**Via**	**Vector/Product**	**Clinical trial code^#^**
CLN2	ERT^*^	Phase 1/2 open label Safety and efficacy	i.c.v.	Recombinant protein	NCT01907087
CLN2	ERT^*^	Open label Long term efficacy	i.c.v.	Recombinant protein	NCT02485899
CLN2	GRT^*^	Safety	i.c.	AAVrh.10CUhCLN2	NCT01161576
CLN3	GRT	Phase 1/2 open-label	i.t.	AT-GTX-502	NCT03770572
CLN6	GRT	Phase 1/2 open-label	i.t.	AT-GTX-501	NCT02725580
CLN6	GRT	Long term efficacy	None	AT-GTX-501-01	NCT04273243
CLN7	GRT	Phase 1 open-label	i.t.	AAV9/CLN7	NCT04737460
CLN3	SRT	Phase 1/2	os	BBDF 101	None

*ERT, Enzyme Replacement Therapy; GRT, Gene Replacement Therapy; SRT, Substrate Reduction Therapy; i.c.v., intracerebralventricular injection; i.c., intracerebral (burr holes) injection; i.t., intrathecal injection*.

* *completed studies*.

# *ClinicalTrials.gov*.

There are ongoing phase 1/2 open label studies to evaluate the safety and efficacy of gene therapy administered intrathecally for CLN3 disease, CLN6 disease and CLN7 disease. These studies have not yet been completed, and the final results are not available (Table 4).

#### Substrate Reduction Therapy

Some preliminary data indicate lysosomal glycosphingolipid accumulation in CLN3 and CLN5 disease. Such findings have suggested an approach using prevention of abnormal lysosomal storage, by interfering with glucosylceramide and ganglioside production using Miglustat a glucosylceramide synthase inhibitor, which is currently used in Niemann-Pick type C disease. This drug is authorized in Europe (but not in USA) for the disease, and beneficial effects of this treatment on juvenile- or adult-onset N-PC were reported on a large cohort of patients (179). Recently it was also shown that trehalose (a disaccharide) promotes lysosomal clearance in storage disease by activation of TFEB, a transcription factor involved in lysosome biogenesis and recruitment (180). By combining the potential benefits of Miglustat and the physiological role of Trehalose a phase 1/2 study is planned using a small number of CLN3 patients using a new recently FDA approved drug (BBDF 101) which contains both components.

#### Storage Dissolution

There was only one study so far, aiming to deplete the endo-lysosomal storage in CLN1 disease by the combined action of phosphocystemaine (which cleaves thioester linkage in palmitoylated protein and N-acetylcysteine a strong anti-oxidant which also cleaves thiosterer linkage). The long term outcome showed only minor subjective benefits for patients, along with some improvements related to EEG pattern and storage dissolution (181).

## Concluding Remarks

NCL are rare, genetically determined, progressive diseases affecting several mammalian species. In humans they affect pre-school and school aged children (or more rarely they start in adulthood) and are defined by clinical criteria supported by pathological features. Amaurosis, seizures, ataxia, behavioral problems, cognitive decline are the major symptoms leading within a relatively short time span to dementia, loss of motor autonomy, blindness and dependence on caregivers. Autofluorescence and selected ultrastructural features of endo-lysosomal storage are the pathological markers detectable both in central and autonomic neurons as well as in several peripheral cells following skin biopsy. Modern diagnosis relies mainly on biochemical (for the NCLs caused by lysosomal enzyme deficiencies) and genetic studies.

Over last two decades dramatic advances in the knowledge of the molecular basis of NCL has been achieved in both humans and several experimental models, but the precise patho-mechanisms leading to cell pathology and neuronal death have not yet been fully elucidated. Nevertheless, a few targeted treatments for the NCLs have become available for human study recently, including enzyme replacement and gene therapies, although only one product is commercially available at present. New pharmacological approaches are foreseen in the near future. The safety and efficacy of such novel treatments are still under scrutiny; there is a conscious effort to ascertain whether new clinical phenotypes may arise as a consequence of these innovative treatments, as recently described in SMA type 1 (182). International collaborations are necessary, recruiting larger cohorts of patients, to generate robust natural history studies to support the regulatory approval process for new drugs (92, 183) and to inform health service delivery.

There is an urgent need to find safe and effective treatments for rare neurodegenerative diseases, such as the NCLs, and mutual agreements between patients, families and advocacy groups, the Health Systems and the pharmaceutical companies are mandatory. With such endeavors, challenges have become evident and will need to be overcome. Examples include ownership and sharing of personal data (natural history data and pre-marketing clinical data), the costs of treatment, the health benefit/cost ratio. These issues are particularly relevant in Europe, where publically funded health systems have to protect and guarantee the privacy of clinical data and, at the same time, have to balance the costs of novel treatments for rare diseases against wider health targets and to guarantee equitable access without discrimination.

## Author Contributions

AS developed the original concept and design of this study. Both authors have made substantial contributions to further modifications of the work, were actively involved in the revision of this manuscript, approved the submitted version and agreed to be personally accountable for the author's own contributions and to ensure any part of the work are appropriately investigated, documented in accordance with the literature, and read and approved the final manuscript.

## Funding

This study was supported by the Italian Ministry of University and Research and by a grant from Fondazione Mariani-Child Neurology to AS. AS is member of the European Reference Network MetabERN.

## Conflict of Interest

The authors declare that the research was conducted in the absence of any commercial or financial relationships that could be construed as a potential conflict of interest.

## Publisher's Note

All claims expressed in this article are solely those of the authors and do not necessarily represent those of their affiliated organizations, or those of the publisher, the editors and the reviewers. Any product that may be evaluated in this article, or claim that may be made by its manufacturer, is not guaranteed or endorsed by the publisher.
