# Supplementary material for: Neuronal Ceroid Lipofuscinosis: The Multifaceted Approach to the Clinical Issues, an Overview
Source: Front Neurol. 2022 Mar 11;13:811686. doi: 10.3389/fneur.2022.811686 (PMC8961688; doi:10.3389/fneur.2022.811686)
Supplement: Supplementary file 1 [file Table_1.DOCX]

**Supplementary Material. Table 1**

**Pathological phenotypes in mammals due to spontaneous mutations in NCL genes, and related references.**

| **animal** | **mutated gene** | **human NCL form** | **reference** |
| --- | --- | --- | --- |
| dog (Dachsund) | *PPT1* | CLN1 disease | Sanders et al, 2010 |
| dog (Dachsund) | *TPP1* | CLN2 disease | Awano et al, 2006 |
| dog (Border Collie) | *CLN5* | CLN5 disease | Mizukami et al, 2012 |
| dog (Golden Retriever) | *CLN5* | CLN5 disease | Gilliam et al, 2015 |
| sheep Borderdale | *CLN5* | CLN5 disease | Frugier et al, 2008 |
| sheep South Hampshire | *CLN6* | CLN6 disease | Tammen et al, 2006 |
| sheep Merino | *CLN6* | CLN6 disease | Tammen et al, 2006 |
| Devon Cattle | *CLN6* | CLN6 disease | Houweling et, 2006 |
| dog (Australian shepherd) | *CLN6* | CLN6 disease | Katz et al, 2011 |
| nclf mouse | *nclf* | CLN6 disease | Wheeler et al, 2006 |
| macaque | *MFSD8* | CLN7 disease | McBride et al, 2018 |
| dog (English setter) | *CLN8* | CLN8 disease | Katz et al, 2005 |
| mnd mouse | *mnd* | CLN8 disease | Ranta et al, 1999 |
| sheep | *CTSD* | CLN10 disease  (connatal form) | Tynnella et al, 2000 |
| dog (American bulldog) | *CTSD* | CLN10 disease | Awano et al, 2006 |
| ???? | *CLN3* | CLN3 disease |  |

Awano, T., Katz, M.L., O'Brien, D.P., Taylor, J.F., Evans, J., Khan, S., Sohar, I., Lobel, P., and Johnson G.S. (2006). [A mutation in the cathepsin D gene (CTSD) in American Bulldogs with neuronal ceroid lipofuscinosis.](https://sslvpn.univr.it/16386934/,DanaInfo=pubmed.ncbi.nlm.nih.gov,SSL+) Mol. Genet. Metab. 87,341-348. doi: 10.1016/j.ymgme.2005.11.005.

Awano, T., Katz, M.L., O'Brien, D.P., Sohar, I., Lobel, P., Coates, J.R., Khan, S., Johnson, G.C., Giger, U., and Johnson G.S. (2006). [A frame shift mutation in canine TPP1 (the ortholog of human CLN2) in a juvenile Dachshund with neuronal ceroid lipofuscinosis.](https://sslvpn.univr.it/16621647/,DanaInfo=pubmed.ncbi.nlm.nih.gov,SSL+) Mol. Genet. Metab. 89, 254-260. doi: 10.1016/j.ymgme.2006.02.016.

Frugier, T., Mitchell, N.L., Tammen, I., Houweling, P.J., Arthur, D.G., Kay, G.W., van Diggelen, O.P., Jolly, R.D., and Palmer, D.N. (2008). [A new large animal model of CLN5 neuronal ceroid lipofuscinosis in Borderdale sheep is caused by a nucleotide substitution at a consensus splice site (c.571+1G>A) leading to excision of exon 3.](https://sslvpn.univr.it/17988881/,DanaInfo=pubmed.ncbi.nlm.nih.gov,SSL+) Neurobiol. Dis. 29, 306-315. doi: 10.1016/j.nbd.2007.09.006.

Gilliam, D., Kolicheski, A., Johnson, G.S., Mhlanga-Mutangadura, T., Taylor, J.F., Schnabel, R.D., and Katz, M.L. (2015). Golden Retriever dogs with neuronal ceroid lipofuscinosis have a two-base-pair deletion and frameshift in CLN5. Mol. Genet. Metab. 115, 101-109.

doi: 10.1016/j.ymgme.2015.04.001.

Houweling, P.J., Cavanagh, J.A., Palmer, D.N., Frugier, T., Mitchell, N.L., Windsor, P.A., Raadsma, H.W., and Tammen, I. (2006). Neuronal ceroid [lipofuscinosis in Devon cattle is caused by a single base duplication (c.662dupG) in the bovine CLN5 gene.](https://sslvpn.univr.it/16935476/,DanaInfo=pubmed.ncbi.nlm.nih.gov,SSL+) Biochim. Biophys. Acta 1762, 890-897. doi: 10.1016/j.bbadis.2006.07.008.

Katz, M.L., Khan, S., Awano, T., Shahid, S.A., Siakotos, A.N., and Johnson, G.S. (2005). [A mutation in the CLN8 gene in English Setter dogs with neuronal ceroid-lipofuscinosis.](https://sslvpn.univr.it/15629147/,DanaInfo=pubmed.ncbi.nlm.nih.gov,SSL+) Biochem. Biophys. Res. Commun. 327, 541-547. doi: 10.1016/j.bbrc.2004.12.038.

Katz, M.L., Farias, F.H., Sanders, D.N., Zeng, R., Khan, S., Johnson, G.S., and O'Brien, D.P. (2011). A missense mutation in canine CLN6 in an Australian shepherd with neuronal ceroid lipofuscinosis. Biomed. Biotechnol. 2011:198042. doi: 10.1155/2011/198042.

McBride, J.L., Neuringer, M., Ferguson, B., Kohama, S.G., Tagge, I.J., Zweig, R.C., Renner, L.M., McGill, T.J., Stoddard, J., Peterson, S., Su, W., Sherman, L.S., Domire, J.S., Ducore, R.M., Colgin, L.M., and Lewis, A.D. (2018). [Discovery of a CLN7 model of Batten disease in non-human primates.](https://sslvpn.univr.it/30048804/,DanaInfo=pubmed.ncbi.nlm.nih.gov,SSL+) Neurobiol. Dis. 119, 65-78. doi: 10.1016/j.nbd.2018.07.013.

Mizukami, K., Kawamichi, T., Koie, H., Tamura, S., Matsunaga, S., Imamoto, S., Saito, M., Hasegawa, D., Matsuki, N., Tamahara, S., Sato, S., Yabuki, A., Chang, H.S., and Yamato, O. (2012). [Neuronal ceroid lipofuscinosis in Border Collie dogs in Japan: clinical and molecular epidemiological study (2000-2011).](https://sslvpn.univr.it/22919312/,DanaInfo=pubmed.ncbi.nlm.nih.gov,SSL+) Scient. World J. 2012:383174. doi: 10.1100/2012/383174.

Ranta, S., Zhang, Y., Ross, B., Lonka, L., Takkunen, E., Messer, A., Sharp, J., Wheeler, R., Kusumi, K., Mole, S.E., Liu, W., Soares, M.B., Bonaldo, M.F., Hirvasniemi, A., de la Chapelle, A., Gilliam, T.C., and Lehesjoki AE. (1999). [The neuronal ceroid lipofuscinoses in human EPMR and mnd mutant mice are associated with mutations in CLN8.](https://sslvpn.univr.it/10508524/,DanaInfo=pubmed.ncbi.nlm.nih.gov,SSL+) Nat. Genet. 23, 233-236. doi: 10.1038/13868.

Sanders, D.N., Farias, F.H., Johnson, G.S., Chiang, V., Cook, J.R., O'Brien, D.P., Hofmann, S.L., Lu, J.Y., and Katz, M.L. (2010). [A mutation in canine PPT1 causes early onset neuronal ceroid lipofuscinosis in a Dachshund.](https://sslvpn.univr.it/20494602/,DanaInfo=pubmed.ncbi.nlm.nih.gov,SSL+) Mol Genet Metab. 100, 349-356. doi: 10.1016/j.ymgme.2010.04.009.

Tammen, I., Houweling, P.J., Frugier, T., Mitchell, N.L., Kay, G.W., Cavanagh, J.A., Cook, R.W., Raadsma, H.W., and Palmer, D.N. (2006). A missense mutation (c.184C>T) in ovine CLN6 causes neuronal ceroid lipofuscinosis in Merino sheep whereas affected South Hampshire sheep have reduced levels of CLN6 mRNA. Biochim. Biophys. Acta 1762, 898-905. doi: 10.1016/j.bbadis.2006.09.004.

Tyynelä, J., Sohar, I., Sleat, D.E., Gin, R.M., Donnelly, R.J., Baumann, M., Haltia, M., and Lobel, P. (2000). [A mutation in the ovine cathepsin D gene causes a congenital lysosomal storage disease with profound neurodegeneration.](https://sslvpn.univr.it/10856224/,DanaInfo=pubmed.ncbi.nlm.nih.gov,SSL+) EMBO J. 19, 2786-2792. doi: 10.1093/emboj/19.12.2786.

Wheeler, R.B., Sharp, J.D., Schultz, R.A., Joslin, J.M., Williams, R.E., and Mole S.E. (2002). The gene mutated in variant late-infantile neuronal ceroid lipofuscinosis (CLN6) and in nclf mutant mice encodes a novel predicted transmembrane protein. Am. J. Hum. Genet. 70, 537-542.

doi: 10.1086/338708.

**Supplementary Material. Table 2.**

**Examples of Omics investigation related to NCL human samples, and related references.**

| **Disease** | **Sample** | **Omics** | **Cell Compartment/Activities** | **References** |
| --- | --- | --- | --- | --- |
| **review** | DNA | genomics |  | Butz et al, 2020 |
| **CLN1/CLN3** | SH-SY5Y cells | proteomics | interactome | Scifo et al, 2015 |
| **CLN1** | human fibroblasts; mouse CNS | transcriptomics,proteomics | growth of plasma projections, mitochondrial and synaptic functions | Tikka et al, 2016 |
| **CLN1** | SH-SY5Y cells | transcriptomics | axonal branching and elongation | Pezzini et al, 2017 |
| **CLN1** | SH-SY5Y cells | transcriptomics | ion channels and neuronal excitability | DeMontis et al, 2020 |
| **CLN1** | human and mouse CNS | comparative proteomics | glial and neuronal cells (mitochondria and cell signalling functions) | Nelvagal et al, 2020 |
| **CLN2** | human CSF | metabolomics | biomarker detection | Sindelar et al, 2018 |
| **CLN1/CLN2/CLN3** | human CSF and brain | proteomics | biomarker detection | Sleat et al, 2017 |
| **CLN1/CLN2/CLN3/**  **CLN5/CLN6/CLN7** | human urine | proteomics | enzyme activity | Iwan et al, 2020 |
| **CLN3** | human skeletal muscle | proteomics | energy metabolism proteins | Santacatterina et al, 2015 |
| **CLN4** | human brain | proteomics | palmitoylated proteins, synapses and lysosomes | Henderson et al, 2016 |
| **CLN5** | SH-SY5Y cells, HeK cells;  mouse CNS | proteomics | mitochondrial dysfunction (mitophagy) | Doccini et al, 2020 |
| **CLN11** | human CNS | transcriptomics, lipidomics | lysosome compartment | Evers et al, 2017 |

Butz, E.S., Chandrachud, U., Mole, S.E., and Cotman, S.L. (2020). [Moving towards a new era of genomics in the neuronal ceroid lipofuscinoses.](https://sslvpn.univr.it/31678159/,DanaInfo=pubmed.ncbi.nlm.nih.gov,SSL+) Biochim. Biophys. Acta Mol. Basis Dis. 1866:165571. doi: 10.1016/j.bbadis.2019.165571.

De Montis, G.C., Pezzini, F., Margari, E., Bianchi, M., Longoni, B., Doccini, S., Lalowski, M.M., Santorelli, F.M., and Simonati, A. (2020). Electrophysiologic profile remodeling via selective suppression of voltage-gated currents by CLN1/PPT1 over expression in human neuronal-like cells. Frontiers in Cellular Neuroscience (Cell Neurophysiology) 14:569598 doi:10.3389/fncel.2020.569598.

Doccini, S., Morani, F., Nesti, C., Pezzini, F., Calza, G., Soliymani, R., Rocchiccioli, S., Kanninen, K.M., Huuskonen, M.T., Baumann, M., Simonati, A., Lalowski, M.M., and Santorelli, F.M. (2020) Proteomic and functional analyses in disease models reveal CLN5 protein involvement in mitochondrial dysfunction. Cell Death Discovery 6:18. [doi.org/10.1038/s41420-020-0250-y](http://doi.org/10.1038/s41420-020-0250-y).

Evers, B.M., [Rodriguez-Navas](https://sslvpn.univr.it/,DanaInfo=pubmed.ncbi.nlm.nih.gov,SSL+?sort=date&term=Rodriguez-Navas+C&cauthor_id=28903038), C., [Rachel J Tesla](https://sslvpn.univr.it/,DanaInfo=pubmed.ncbi.nlm.nih.gov,SSL+?sort=date&term=Tesla+RJ&cauthor_id=28903038), R.J., [Prange-Kiel](https://sslvpn.univr.it/,DanaInfo=pubmed.ncbi.nlm.nih.gov,SSL+?sort=date&term=Prange-Kiel+J&cauthor_id=28903038), J., [Wasser](https://sslvpn.univr.it/,DanaInfo=pubmed.ncbi.nlm.nih.gov,SSL+?sort=date&term=Wasser+CR&cauthor_id=28903038), C.R., [Yoo](https://sslvpn.univr.it/,DanaInfo=pubmed.ncbi.nlm.nih.gov,SSL+?sort=date&term=Yoo+KS&cauthor_id=28903038),, K.S., [McDonald](https://sslvpn.univr.it/,DanaInfo=pubmed.ncbi.nlm.nih.gov,SSL+?sort=date&term=McDonald+J&cauthor_id=28903038), J., [Cenik](https://sslvpn.univr.it/,DanaInfo=pubmed.ncbi.nlm.nih.gov,SSL+?sort=date&term=Cenik+B&cauthor_id=28903038), B., [Ravenscroft](https://sslvpn.univr.it/,DanaInfo=pubmed.ncbi.nlm.nih.gov,SSL+?sort=date&term=Ravenscroft+TA&cauthor_id=28903038), T.A., [Plattner](https://sslvpn.univr.it/,DanaInfo=pubmed.ncbi.nlm.nih.gov,SSL+?sort=date&term=Plattner+F&cauthor_id=28903038), F., [Rademakers](https://sslvpn.univr.it/,DanaInfo=pubmed.ncbi.nlm.nih.gov,SSL+?sort=date&term=Rademakers+R&cauthor_id=28903038), R., [Yu](https://sslvpn.univr.it/,DanaInfo=pubmed.ncbi.nlm.nih.gov,SSL+?sort=date&term=Yu+G&cauthor_id=28903038), G., [White 3rd](https://sslvpn.univr.it/,DanaInfo=pubmed.ncbi.nlm.nih.gov,SSL+?sort=date&term=White+CL+3rd&cauthor_id=28903038), C.L., [and Herz](https://sslvpn.univr.it/,DanaInfo=pubmed.ncbi.nlm.nih.gov,SSL+?sort=date&term=Herz+J&cauthor_id=28903038), J.(2017). Lipidomic and Transcriptomic Basis of Lysosomal Dysfunction in Progranulin Deficiency**.** Cell Rep. 20, 2565-2574. doi: 10.1016/j.celrep.2017.08.056.

Henderson, M.X., Wirak, G.S., Zhang, Y.Q., Dai, F., Ginsberg, S.D., Dolzhanskaya, N., Staropoli, J.F., Nijssen, P.C., Lam, T.T., Roth, A.F., Davis, N.G., Dawson, G., Velinov, M., and Chandra, S.S. (2016). Neuronal ceroid lipofuscinosis with DNAJC5/CSPalpha mutation has PPT1 pathology and exhibit aberrant protein palmitoylation. Acta Neuropathol. 131, 621-637.

doi: 10.1007/s00401-015-1512-2.

[Iwan](https://sslvpn.univr.it/,DanaInfo=pubmed.ncbi.nlm.nih.gov,SSL+?sort=date&term=Iwan+K&cauthor_id=33532713), K. [Robert Clayton](https://sslvpn.univr.it/,DanaInfo=pubmed.ncbi.nlm.nih.gov,SSL+?sort=date&term=Clayton+R&cauthor_id=33532713), R., [Mills](https://sslvpn.univr.it/,DanaInfo=pubmed.ncbi.nlm.nih.gov,SSL+?sort=date&term=Mills+P&cauthor_id=33532713), P., [Csanyi](https://sslvpn.univr.it/,DanaInfo=pubmed.ncbi.nlm.nih.gov,SSL+?sort=date&term=Csanyi+B&cauthor_id=33532713), B., [Gissen](https://sslvpn.univr.it/,DanaInfo=pubmed.ncbi.nlm.nih.gov,SSL+?sort=date&term=Gissen+P&cauthor_id=33532713), P., [Mole](https://sslvpn.univr.it/,DanaInfo=pubmed.ncbi.nlm.nih.gov,SSL+?sort=date&term=Mole+SE&cauthor_id=33532713), S.E., [Palmer](https://sslvpn.univr.it/,DanaInfo=pubmed.ncbi.nlm.nih.gov,SSL+?sort=date&term=Palmer+DN&cauthor_id=33532713), D.N., [Mills](https://sslvpn.univr.it/,DanaInfo=pubmed.ncbi.nlm.nih.gov,SSL+?sort=date&term=Mills+K&cauthor_id=33532713), K., [and Heywood](https://sslvpn.univr.it/,DanaInfo=pubmed.ncbi.nlm.nih.gov,SSL+?sort=date&term=Heywood+WE&cauthor_id=33532713), W.E.. (2020). Urine proteomics analysis of patients with neuronal ceroid lipofuscinoses**.** iScience 24:102020. doi: 10.1016/j.isci.2020.102020.

Nelvagal, H.R., Hurtado, M.L., Eaton, S.L., Kline, R.A., Lamont, D.J., Sands, M.S., Wishart, T.M., and Cooper, J.D. (2020). [Comparative proteomic profiling reveals mechanisms for early spinal cord vulnerability in CLN1 disease.](https://sslvpn.univr.it/32938982/,DanaInfo=pubmed.ncbi.nlm.nih.gov,SSL+) Sci Rep. 10:15157. doi: 10.1038/s41598-020-72075-7.

Pezzini. F., Bianchi, M., Benfatto, S., Griggio, F., Doccini, S., Carrozzo, R., Dapkunas, A., Delledonne, M., Santorelli, F.M., Lalowski, M., and Simonati, A. (2017). The networks of genes encoding palmitoylated proteins in axonal and synaptic compartments are affected in PPT1 overexpressing neuronal-like cells. Front. Mol. Neurosci. 10:266. doi: 10.3389/fnmol.2017.00266.

[Santacatterina](https://sslvpn.univr.it/,DanaInfo=pubmed.ncbi.nlm.nih.gov,SSL+?sort=date&term=Santacatterina+F&cauthor_id=25880557), F., [Chamorro](https://sslvpn.univr.it/,DanaInfo=pubmed.ncbi.nlm.nih.gov,SSL+?sort=date&term=Chamorro+M&cauthor_id=25880557), M., C., [Navarro](https://sslvpn.univr.it/,DanaInfo=pubmed.ncbi.nlm.nih.gov,SSL+?sort=date&term=Navarro+C&cauthor_id=25880557), C., [Martín](https://sslvpn.univr.it/,DanaInfo=pubmed.ncbi.nlm.nih.gov,SSL+?sort=date&term=Mart%C3%ADn+MA&cauthor_id=25880557), M.A., [Cuezva](https://sslvpn.univr.it/,DanaInfo=pubmed.ncbi.nlm.nih.gov,SSL+?sort=date&term=Cuezva+JM&cauthor_id=25880557), J.M., and [Sánchez-Aragó](https://sslvpn.univr.it/,DanaInfo=pubmed.ncbi.nlm.nih.gov,SSL+?sort=date&term=S%C3%A1nchez-Arag%C3%B3+M&cauthor_id=25880557), M. (2015). Quantitative analysis of proteins of metabolism by reverse phase protein microarrays identifies potential biomarkers of rare neuromuscular diseases**.** J. Transl. Med. 13:65.

doi: 10.1186/s12967-015-0424-1.

Scifo, E., Szwajda. A., Soliymani, R., Pezzini, F., Bianchi, M., Dapkunas, A., Dębski, J., Uusi-Rauva, K., Dadlez. M., Gingras, A.C., Tyynelä, J., Simonati, A., Jalanko, A., Baumann, M.H., and Lalowski, M. (2015). [Proteomic analysis of the palmitoyl protein thioesterase 1 interactome in SH-SY5Y human neuroblastoma cells.](https://sslvpn.univr.it/25865307/,DanaInfo=pubmed.ncbi.nlm.nih.gov,SSL+) J. Proteomics 123, 42-53. doi: 10.1016/j.jprot.2015.03.038.

[Sindelar](https://sslvpn.univr.it/,DanaInfo=pubmed.ncbi.nlm.nih.gov,SSL+?sort=date&term=Sindelar+M&cauthor_id=30323181), M. [Dyke](https://sslvpn.univr.it/,DanaInfo=pubmed.ncbi.nlm.nih.gov,SSL+?sort=date&term=Dyke+JP&cauthor_id=30323181), J.P. [Deeb](https://sslvpn.univr.it/,DanaInfo=pubmed.ncbi.nlm.nih.gov,SSL+?sort=date&term=Deeb+RS&cauthor_id=30323181), R.S., [Sondhi](https://sslvpn.univr.it/,DanaInfo=pubmed.ncbi.nlm.nih.gov,SSL+?sort=date&term=Sondhi+D&cauthor_id=30323181), D.,  [Kaminsky](https://sslvpn.univr.it/,DanaInfo=pubmed.ncbi.nlm.nih.gov,SSL+?sort=date&term=Kaminsky+SM&cauthor_id=30323181), S.M.,  [Kosofsky](https://sslvpn.univr.it/,DanaInfo=pubmed.ncbi.nlm.nih.gov,SSL+?sort=date&term=Kosofsky+BE&cauthor_id=30323181),  B.E., [Ballon](https://sslvpn.univr.it/,DanaInfo=pubmed.ncbi.nlm.nih.gov,SSL+?sort=date&term=Ballon+DJ&cauthor_id=30323181), D.J. , R.G., and Gross, S.S. (2018). Untargeted Metabolite Profiling of Cerebrospinal Fluid Uncovers Biomarkers for Severity of Late Infantile Neuronal Ceroid Lipofuscinosis (CLN2, Batten Disease)**.** Sci. Rep. 8:15229.  doi: 10.1038/s41598-018-33449-0.

Sleat, D.E., Tannous, A., Sohar, I., Wiseman, J.A., Zheng, H., Qian, M., Zhao, C., Xin, W., Barone, R., Sims, K.B., Moore, D.F., and Lobel, P. (2017). Proteomic Analysis of Brain and Cerebrospinal Fluid from the Three Major Forms of Neuronal Ceroid Lipofuscinosis Reveals Potential Biomarkers J. Proteome Res. 16, 3787-3804. doi: 10.1021/acs.jproteome.7b00460.

Tikka, S., Monogioudi, E., Gotsopoulos, A., Soliymani, R., Pezzini, F., Scifo, E., Uusi-Rauva, K., Tyynelä, J., Baumann, M., Jalanko, A., Simonati, A., and Lalowski, M. (2016) Proteomic profiling in the brain of CLN1 disease model reveals affected functional modules. NeuroMolecular Medicine 18, 109-133. doi: 10.1007/s12017-015-8382-6.
